# Analysis of the actor-partner interdependence mediation model for self-disclosure between social support and benefit finding among stroke patients and their caregivers

**DOI:** 10.3389/fpubh.2026.1890113

**Published:** 2026-10-02

**Authors:** Susu Zhu, Ning Ban, Xiao Miao, Meng Guo, Liang Ma

**Affiliations:** 1 Department of Nursing, The Affiliated Lianyungang Hospital of Xuzhou Medical University, Lianyungang, China; 2 Department of Neurosurgery, The Affiliated Lianyungang Hospital of Xuzhou Medical University, Lianyungang, China; 3 Department of Neurocritical Care, The Affiliated Lianyungang Hospital of Xuzhou Medical University, Lianyungang, China

**Keywords:** actor-partner interdependence mediating model, benefit finding, self-disclosure, social support, stroke

## Abstract

**Objective:**

Stroke is an acute, sudden-onset condition that constitutes an acute stressful event for both patients and caregivers, exerting a significant psychological impact. Based on the actor-partner interdependence mediating model, this study examines the influence of social support on benefit finding among stroke patients and their caregivers and investigates whether self-disclosure mediates this relationship.

**Methods:**

This study is a cross-sectional study. Using convenience sampling, 278 pairs of stroke patients and their caregivers were selected from a Grade III Level A hospital in Lianyungang City, Jiangsu Province, China, as the study subjects. The assessment tools included a general information questionnaire, the Social Support Rating Scale, the Distress Disclosure Index, and the Benefit Finding Scale. Data analysis was performed using SPSS 27.0 and Mplus 8.3 software, and a 95% confidence interval for the indirect path was estimated using bootstrap resampling with 5,000 repetitions to correct for bias.

**Results:**

Regarding actor effects, the indirect paths from patients’ and caregivers’ own social support to benefit finding via self-disclosure were both statistically significant (*β* = 0.106, 95% *CI* = 0.055–0.156; *β* = 0.072, 95% *CI* = 0.029–0.115) Regarding the partner effect, the indirect path from the caregivers’ social support to the patients’ benefit finding via the patients’ self-disclosure was statistically significant (*β* = 0.058, 95% *CI* = 0.015–0.102).

**Conclusion:**

The results indicate an actor-partner interdependence mediating effect among social support, self-disclosure, and benefit finding among stroke patients and their caregivers. This provides support for enhancing benefit finding among stroke patients and their caregivers; healthcare professionals can facilitate this by expanding the social support networks of patients and caregivers and encouraging them to express their inner thoughts, thereby enhancing benefit finding during the course of treatment.

## 1 Introduction

Stroke is an acute cerebrovascular disorder caused by cerebral vascular lesions. It is classified into ischemic stroke and hemorrhagic stroke (1). It is characterized by five key features: high incidence, high recurrence rate, high disability rate, high mortality rate, and high economic burden (2). It is a leading cause of death and disability worldwide (3). Studies indicate that the recurrence rate within 1 year of onset is 7.48%, and within 5 years is 41% (4). Post-stroke patients may suffer from various neurological complications, such as motor impairments, cognitive impairments, swallowing difficulties, and fatigue (5). The probability of post-stroke cognitive impairment occurring within the first year after a stroke is as high as 60%, affecting patients’ functional abilities and quality of life and preventing them from maintaining normal work, independent living, and social interactions (6).

Stroke can also lead to psychological disorders in patients. The most common psychological issues following a stroke are depression, anxiety, and stress. Studies indicate (7, 8) that the incidence of depression ranges from 9% to 21% within 3 months to 1 year after a stroke, with a cumulative incidence ranging from 21% to 55% within the first year. The lifetime prevalence of depression is 27%, while the prevalence rates for anxiety and stress are 52.9% and 68%, respectively. Studies indicate that stroke not only affects patients’ quality of life but also impacts that of their caregivers (9). Caring for a patient is a complex and demanding task; over 50% of caregivers experience physical exhaustion and psychological distress due to the excessive burden of care (10). Reported prevalence rates of depression and anxiety among caregivers range from 30% to 45% and 20% to 50% (9), respectively. Thus, it is evident that stroke not only severely compromises patients’ physical and mental health and quality of life but also imposes a heavy physical and psychological burden on their caregivers.

Researchers have found that when individuals experience extremely severe traumatic stressors, they naturally experience negative emotions such as anxiety and depression; however, these are not the only emotions involved. Such experiences may also hold the potential for a shift toward positive psychological states, such as post-traumatic growth and benefit finding (11).

Benefit finding is a psychological process in which individuals, following adverse experiences such as trauma, illness, or disaster, perceive and identify the positive changes, gains, or personal growth resulting from such events (12). Research indicates that benefit finding enhances the quality of life for both patients and caregivers (13, 14) and can alleviate depressive symptoms (15). Another study noted that while benefit finding does not directly improve patients’ quality of life, it helps reduce the negative impacts caused by the burden of symptoms (16). For caregivers, benefit finding can enhance their caregiving capacity, thereby improving patient recovery (17).

Lyons et al. (18) proposed that disease management is a dyadic phenomenon, with patients and caregivers forming an interdependent dyad. The theory of interpersonal interdependence (19) posits that an individual’s behavioral outcomes influence both their own and others’ behaviors. This theory holds particularly significant practical implications in the context of stroke care. Research indicates (20) that 75% of stroke survivors experience impaired activities of daily living due to residual functional impairments and are largely dependent on the support of caregivers; the two form a close dyad. Research has confirmed (7) that depression and anxiety in stroke patients and their caregivers not only affect their own quality of life but also impact the quality of life of the other party. Therefore, it is necessary to study stroke patients and their caregivers as a unified whole to examine the interactions between them. While there are currently some studies on the actor-partner effect among stroke patients and their caregivers (21, 22), most existing studies on benefit finding have been observed from the perspective of either the patient alone (23) or the caregiver alone (24). One study found (25) that the positive psychological capital and benefit finding among stroke patients and their caregivers exhibit an actor-partner effect, suggesting that the benefit finding by stroke patients or caregivers does not occur in isolation. However, existing studies have not been able to fully elucidate the dyadic interaction mechanism of benefit finding between stroke patients and their caregivers; therefore, further targeted research based on the actor-partner interdependence mediating model is still needed.

Social support refers to the material and emotional assistance individuals receive from others through social interactions; it constitutes an available external resource. Both patients and caregivers require greater social support when coping with illness (26). Research indicates (27) that social support is a factor influencing the perception of benefits, and the two are positively correlated. The higher the level of social support, the higher the level of perceived benefits (28).

Self-disclosure refers to the process by which an individual, during interactions with others, voluntarily and proactively shares their true thoughts, as well as their innermost emotions and feelings, with others (29). Self-disclosure (30) enables individuals to better manage stressors, reduces the physical and mental exhaustion caused by the suppression of negative emotions, and has a positive impact on an individual’s health. Research has found (31) that perceived social support is positively correlated with self-disclosure; furthermore, studies have shown (32) that higher levels of self-disclosure are associated with higher levels of benefit finding. Based on the above analysis, we hypothesize that among stroke patients and their caregivers, social support can promote self-disclosure, which in turn helps them identify positive values in adversity and enhance benefit finding; that is, social support is associated with benefit finding through the mediating effect of self-disclosure.

This study draws on the stress process theory proposed by Taylor and Stanton (33) to examine the influence of external social support on benefit finding. This theory posits that when faced with stressful or traumatic events, individuals adapt to environmental changes by mobilizing and activating internal psychological resources such as optimism, a sense of control, and self-esteem as well as social support, a key external resource. Through these means, they adopt positive coping strategies to overcome difficulties and mitigate the negative physical and psychological impacts. In the stress adaptation process, self-disclosure is a proactive coping behavior that individuals engage in by relying on external support resources. Adequate social support can encourage individuals to express their emotions and stress, thereby reframing the positive meaning of stressful events and enhancing their ability to identify benefits. Based on this, in dyads consisting of stroke patients and their caregivers, we hypothesize that social support, self-disclosure, and benefit finding are mutually correlated; that social support exerts both direct and indirect effects on benefit finding; and that self-disclosure mediates these relationships. See Figure 1.

**Figure 1:**
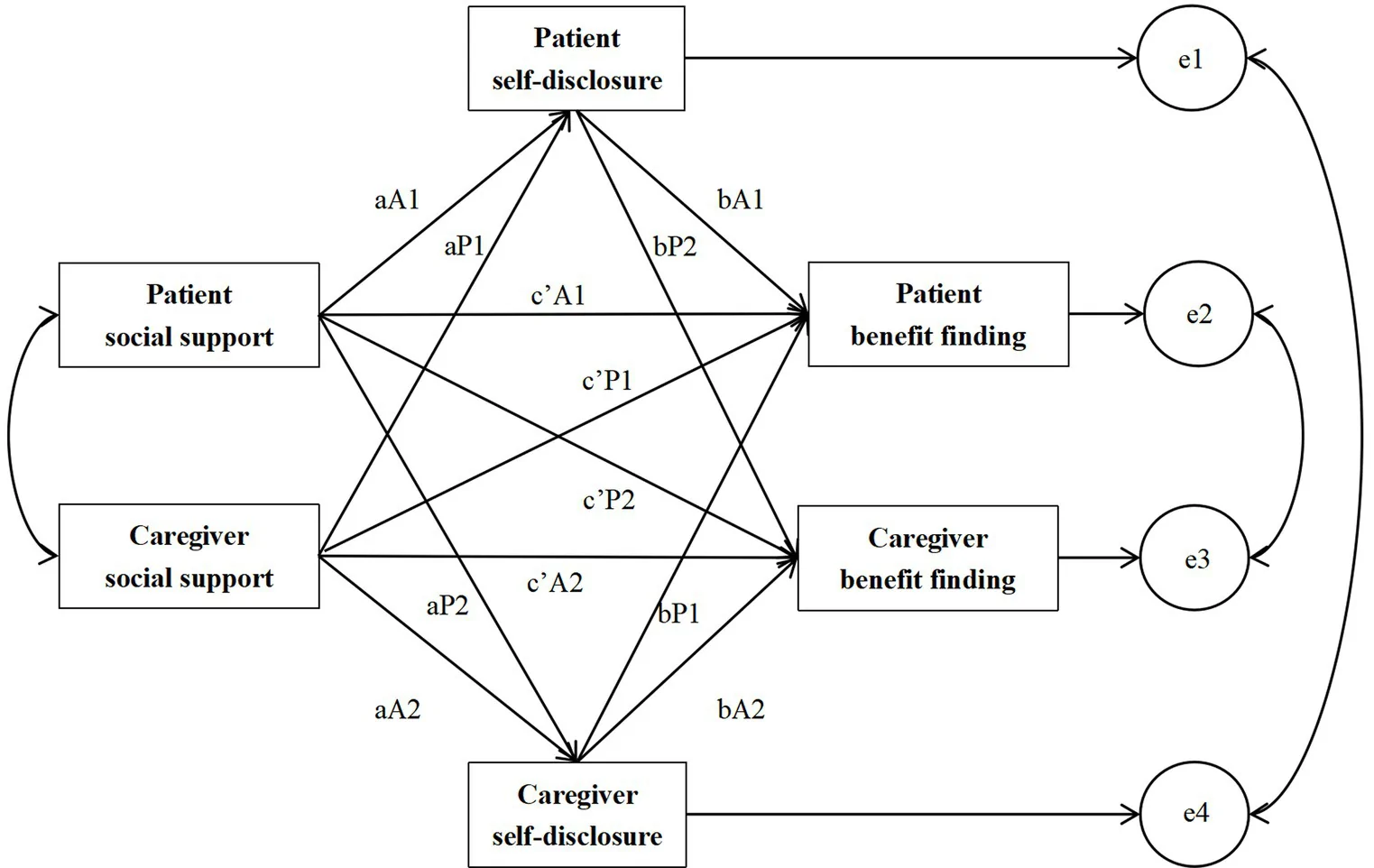
Basic model structure.

## 2 Methods

### 2.1 Study participants

This study is a cross-sectional study that adopted a convenience sampling method. The study population consisted of stroke patients and their caregivers who were hospitalized in the Department of Neurology and Neurosurgery at a Grade III Level A hospital in Lianyungang City between August 2024 and April 2025. Data were collected through face-to-face questionnaire surveys. Inclusion criteria for patients were: (1) meeting the clinical diagnostic requirements outlined in the Chinese Guidelines for the Diagnosis and Treatment of Acute Ischemic Stroke (2018 Edition) (34) and the Chinese Guidelines for the Diagnosis and Treatment of Intracerebral Hemorrhage (2019 Edition) (35); (2) being at least 18 years of age; (3) Stable condition and normal vital signs following treatment; (4) The patient was alert and had no cognitive or communication impairments; (5) Voluntarily signed an informed consent form and agreed to participate in this study. Exclusion criteria: (1) Presence of other life-threatening serious illnesses, such as malignant tumors or multi-organ failure; (2) History of mental illness. Inclusion criteria for caregivers: (1) Aged 18 years or older; (2) Must be the patient’s spouse, parent, child, or other family member; (3) Must be the patient’s primary caregiver, i.e., providing care for more than 4 h daily and undertaking the primary caregiving responsibilities; (4) Must voluntarily sign the informed consent form and agree to participate in this study. Exclusion criteria for caregivers: (1) History of mental illness or communication impairments; (2) Receiving compensation; (3) Recent history of severe psychological trauma. The online tool APIMPowerR (36) was used to estimate the required minimum sample size, assuming a statistical power (1-*β*) of 0.9 and a two-sided *α* of 0.05; a medium effect size of 0.2 was specified for the actor-partner effects of patients and caregivers, respectively. The minimum sample size was calculated to be 264 pairs. Taking into account a 10% non-response rate, the required sample size was 293 pairs. Ultimately, 293 questionnaires were distributed, and 278 were returned, resulting in a response rate of 94.9%. This study has been approved by the hospital’s Institutional Review Board (KY-20240806001-01), all participants provided written informed consent. To protect participant privacy, anonymous questionnaires were administered, and no personally identifiable information (e.g., names or contact details) was collected. A unique sequential study identification code was allocated to each dyad during questionnaire distribution for data management.

### 2.2 Research tools

#### 2.2.1 General information questionnaire

This questionnaire was designed by the researchers and consists of two parts. The section on patient demographics includes gender, age, marital status, educational background, and monthly income, while the section on disease-related information includes stroke type, number of strokes, and family history. The section on caregiver demographics includes gender, age, marital status, and relationship to the patient.

#### 2.2.2 The social support rating scale

SSRS was developed by Xiao (37) in 1986. It consists of 3 dimensions and 10 items, with a total score ranging from 12 to 66 points. A score of 22 or below indicates a low level of social support, 22–44 indicates a moderate level, and 44 or above indicates a high level. The Cronbach’s *α* coefficients for stroke patients and caregivers were 0.708 and 0.707, respectively.

#### 2.2.3 The distress disclosure index

DDI was developed by Kahn et al. (38) and translated and revised by Li (39). It consists of a single dimension with 12 items and uses a 5-point Likert scale ranging from “Strongly Disagree” to “Strongly Agree,” scored from 1 to 5, respectively. Items 2, 4, 5, 8, 9, and 10 are reverse-scored. The total score ranges from 12 to 60 points; a higher total score indicates a higher level of self-disclosure. Scores of 12–29 indicate low-level disclosure, 30–44 indicate moderate-level disclosure, and 45–60 indicate high-level disclosure. In this study, the Cronbach’s *α* coefficients for stroke patients and their caregivers were 0.913 and 0.870, respectively.

#### 2.2.4 The benefit finding scale

BFS was originally developed by Antoni et al. (40), revised by Weaver et al. (41), and adapted into Chinese by Liu et al. (42) for use with cancer patients. It consists of 22 items across 6 dimensions, with a total score ranging from 22 to 110. Liu et al. (43) applied this scale to stroke patients, and the Cronbach’s *α* coefficient was 0.946. Bian et al. (44) revised the Chinese version of the BFS for use with caregivers, which comprises 5 dimensions and 22 items, with a total score ranging from 22 to 110. Wang et al. (45) found that this scale has good reliability and validity among caregivers of stroke patients. In this study, the Cronbach’s *α* coefficients for stroke patients and their caregivers were 0.730 and 0.799, respectively.

### 2.3 Quality assurance

The study was conducted by two trained nursing graduate students who distributed paper questionnaires on-site. Before the survey began, the researchers explained the purpose and significance of the study to the participants and obtained their informed consent. When completing the questionnaire, researchers instructed patients and caregivers to fill out the forms separately and independently, without interfering with one another. For participants unable to complete the questionnaire on their own, the researcher read aloud the questionnaire content and options in a neutral tone, question by question. After the participant made their own independent selection, the researcher marked the corresponding answer on their behalf to minimize response bias as much as possible. Once the questionnaires were completed, the researchers collected them immediately and verified the completion status of each questionnaire to ensure its integrity. Each pair of patient and caregiver questionnaires was assigned a uniform number (e.g., patient P001, caregiver C001) to ensure the accurate linking of paired data. We distributed 293 pairs of questionnaires for this survey, and 286 pairs were returned. Of these, 8 pairs were excluded entirely due to a high number of missing responses (a missing-item rate exceeding 20%) and a pattern of selecting the same option for every item. There were no instances where only one questionnaire in a pair was excluded while the other was retained. The 278 pairs questionnaire pairs ultimately included in the analysis had no missing items; therefore, no methods for imputing missing values, such as multiple imputation, were employed. All statistical analyses were conducted based on the complete dataset. See Figure 2.

**Figure 2:**
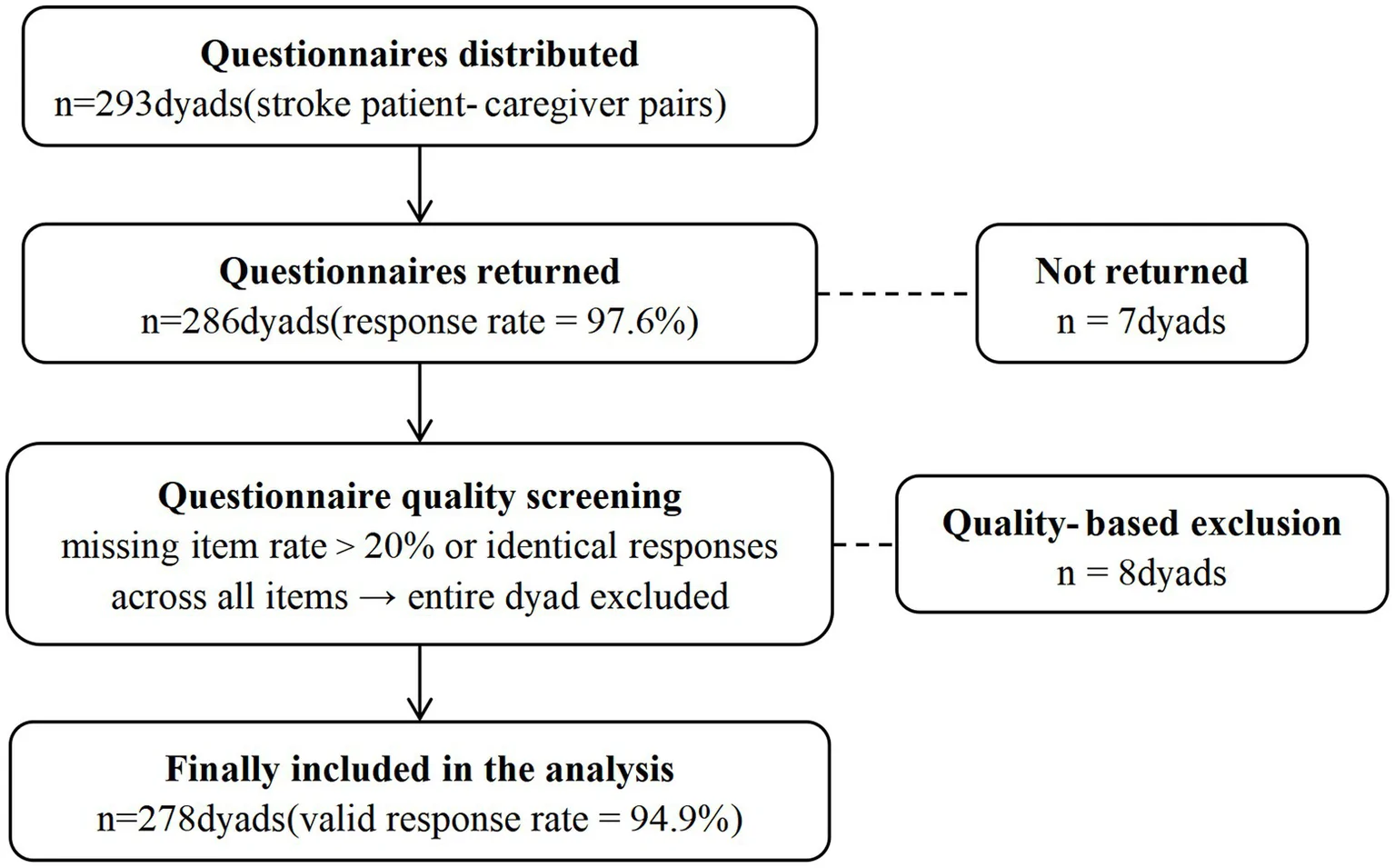
Flow diagram of study participants.

### 2.4 Statistical analysis

Statistical analysis of the data was performed using SPSS 27.0 software. Continuous variables that followed a normal distribution are expressed as mean ± standard deviation ( $\bar{x}\pm s$ ); categorical variables are expressed as frequency and percentage. The scores on the stroke patient and caregiver scales were analyzed using a paired *t*-test because patients and caregivers form a binary paired relationship, their scores are intrinsically correlated, and the difference between their scores follows a normal distribution, thereby satisfying the prerequisites for the paired *t*-test. A normality test was conducted on the variables; since most variables did not follow a normal distribution, Spearman’s rank correlation analysis was used to examine the correlations among the variables. Mplus 8.3 was used to construct an actor-partner interdependence mediating model, and the mediating model was tested using Bootstrap analysis. The significance level was set at *α* = 0.05. Effect sizes were estimated using the maximum likelihood method, and confidence intervals were estimated using Bootstrap sampling with a sample size of 5,000; a 95% confidence interval that does not include 0 was interpreted as evidence of mediation. The model fit indices and their acceptable ranges are as follows: χ^2^/df (degrees of freedom) < 3, CFI (Comparative Fit Index) ≥ 0.9, TLI (Tucker-Lewis Index) ≥ 0.9, and RMSEA (Root Mean Square Error of Approximation) ≤ 0.08.

## 3 Results

### 3.1 Demographic characteristics

Among the 278 patients, 64% were male, and 50.7% were aged 60 or older. Among the 278 caregivers, 57.2% were female, and 70.5% were under the age of 60. See Table 1 for details.

**Table 1:** Demographic information of patients and caregivers (*n* = 278).

Variables	Patients *N* (%)	Caregivers *N* (%)
Gender		
Male	178 (64.0)	119 (42.8)
Female	100 (36.0)	159 (57.2)
Age		
<50	38 (13.7)	138 (49.6)
50–59	99 (35.6)	58 (20.9)
60–69	121 (43.5)	73 (26.3)
≥70	20 (7.2)	9 (3.2)
Marriage		
Married	258 (92.8)	236 (84.9)
Single	3 (1.1)	36 (12.9)
Divorced	5 (1.8)	6 (2.2)
Widowed	12 (4.3)	—
Culture		
Primary School	104 (37.4)	61 (21.9)
Middle School	99 (35.6)	95 (34.2)
High School and Vocational School	60 (21.6)	71 (25.5)
Bachelor’s Degree and Above	15 (5.4)	51 (18.3)
Occupation		
Employed	42 (15.1)	121 (43.5)
Freelancer	54 (19.4)	25 (9.0)
Unemployed	112 (40.3)	80 (28.8)
Retired	70 (25.2)	52 (18.7)
Residence		
City	77 (27.7)	101 (36.3)
Town	62 (22.3)	62 (22.3)
Rural	139 (50.0)	115 (41.4)
Monthly income		
<2,000	73 (26.3)	47 (16.9)
2,001–3,000	54 (19.4)	40 (14.4)
3,001–4,000	52 (18.7)	53 (19.1)
>4,000	99 (35.6)	138 (49.6)
Medical insurance type		
Self-paid	6 (2.2)	—
New rural cooperative medical scheme	167 (60.1)	—
Urban and rural resident medical insurance	16 (5.8)	—
Employee medical insurance	89 (32.0)	—
Types of stroke		
Hemorrhagic stroke	15 (5.4)	—
Ischemic stroke	263 (94.6)	—
Number of strokes		
First release	198 (71.2)	—
2 times	59 (21.2)	—
3 times	14 (5.0)	—
4 times or more	7 (2.5)	—
Family history		
Yes	82 (29.5)	—
None	196 (70.5)	—
Complications		
None	57 (20.5)	—
Yes	221 (79.5)	—
Barthel Index		
0–40	25 (9.0)	—
41–60	51 (18.3)	—
61–99	190 (68.3)	—
100	12 (4.3)	—
Braden Score		
13–14	6 (2.2)	—
15–18	24 (8.6)	—
19–23	248 (89.2)	—
NIHSS score		
Normal	166 (59.7)	—
Mild stroke	82 (29.5)	—
Moderate stroke	30 (10.8)	—
mRS score		
Level 0: Completely asymptomatic	171 (61.5)	—
Level 1: Symptomatic but without disability	47 (16.9)	—
Level 2: Mild disability	29 (10.4)	—
Level 3: Moderate disability	23 (8.3)	—
Level 4: Severe disability	8 (2.9)	—
Length of hospital stay		
1–5 days	20 (7.2)	—
5–10 days	210 (75.5)	—
11–15 days	38 (13.7)	—
≥16 days	10 (3.6)	—
Relationship with Patients		
Spouse	—	151 (54.3)
Children	—	97 (34.9)
Parents	—	5 (1.8)
Siblings	—	12 (4.3)
Other	—	13 (4.7)
Daily care hours		
4–8 h	—	42 (15.1)
8–12 h	—	48 (17.3)
12–16 h	—	19 (6.8)
≥16 h	—	169 (60.8)
Whether having other co-caregivers		
None	—	132 (47.5)
1	—	73 (26.3)
2	—	57 (20.5)
3 and above	—	16 (5.8)
Having or no caregiving experience		
Yes	—	112 (40.3)
None	—	166 (59.7)

### 3.2 A comparison of the scores for social support, self-disclosure, and benefit finding among stroke patients and their caregivers

Differences in social support, self-disclosure, and benefit-finding scores between stroke patients and their caregivers were analyzed using a paired *t*-test. The results showed that the social support score for stroke patients was (43.73 ± 5.60) points, while that for caregivers was (41.35 ± 6.00) points. The social support score for stroke patients was higher than that of their caregivers, and the difference was statistically significant (*t* = 5.481, *p* < 0.001). The self-disclosure score for stroke patients was (35.98 ± 8.38) points, while that for caregivers was (37.38 ± 6.95) points; stroke patients’ self-disclosure scores were lower than those of their caregivers, and the difference was statistically significant (*t* = −2.418, *p* = 0.016). The benefit finding scores for stroke patients were (65.20 ± 8.41) points, while those for caregivers were (71.57 ± 9.90) points. The benefit finding scores for stroke patients were significantly lower than those of their caregivers, and the difference was statistically significant (*t* = −10.135, *p* < 0.001), as shown in Table 2.

**Table 2:** Comparison of scores and differences in social support, self-disclosure, and benefit finding among stroke patients and their caregivers (*n* = 278).

Variables	Score range	Patients	Caregivers	*t*	*p*
Social support	12–66	43.73 ± 5.60	41.35 ± 6.00	5.481	<0.001
Self-disclosure	12–60	35.98 ± 8.38	37.38 ± 6.95	−2.418	0.016
Benefit finding	22–110	65.20 ± 8.41	71.57 ± 9.90	−10.135	<0.001

### 3.3 Correlation analysis of social support, self-disclosure, and benefit finding among stroke patients and caregivers

Social support among stroke patients and caregivers was positively correlated with both self-disclosure and benefit finding (all *p* < 0.01), and self-disclosure among patients and caregivers was positively correlated with benefit finding (all *p* < 0.01), as shown in Table 3.

**Table 3:** Correlation analysis of social support, self-disclosure, and benefit finding among stroke patients and their caregivers (*n* = 278, *r*).

Variables	Patients			Caregivers		
	Social support	Self-disclosure	Benefit finding	Social support	Self-disclosure	Benefit finding
Patients						
Social Support	1					
Self-Disclosure	0.361**	1				
Benefit Finding	0.319**	0.410**	1			
Caregivers						
Social Support	0.243**	0.252**	0.246**	1		
Self-Disclosure	0.140*	0.202**	0.032	0.307**	1	
Benefit Finding	0.345**	0.309**	0.345**	0.315**	0.338**	1

### 3.4 An actor-partner interdependence mediating model of social support, self-disclosure, and benefit finding among stroke patients and caregivers

An actor-partner interdependence mediating model was constructed with social support among stroke patients and caregivers as the predictor variable, self-disclosure as the mediator variable, and benefit finding as the outcome variable, resulting in the model shown in Figure 3. The model fit is good, with the following fit indices: χ^2^/df = 0.005, RMSEA = 0.000, CFI = 1.000, and TLI = 1.058.

**Figure 3:**
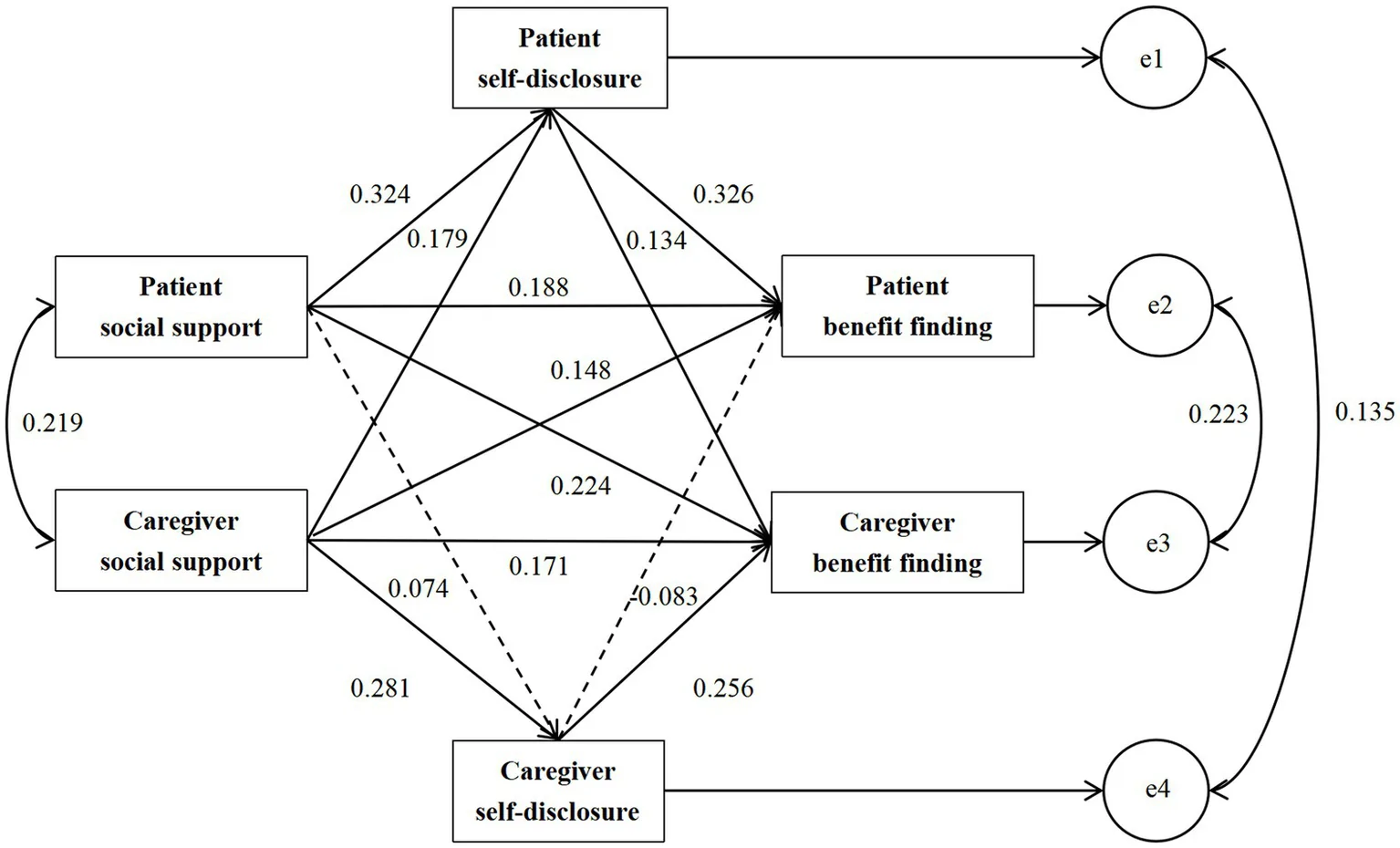
Actor-partner interdependence mediation model of social support, self-disclosure, and benefit finding for patients and caregivers.

In terms of actor effects, social support for stroke patients and their caregivers was found to have a positive predictive effect on benefit finding (*β* = 0.188, *p* < 0.001; *β* = 0.171, *p* < 0.001); social support for stroke patients and their caregivers was also found to have a positive predictive effect on their self-disclosure (*β* = 0.324, *p* < 0.001; *β* = 0.281, *p* = 0.003); self-disclosure by stroke patients and caregivers also had a positive predictive effect on benefit finding (*β* = 0.326, *p* < 0.001; *β* = 0.256, *p* < 0.001); From the object effect perspective, patients’ social support and self-disclosure had a positive predictive effect on caregivers’ benefit finding (*β* = 0.224, *p* < 0.001; *β* = 0.134, *p* = 0.031); The caregiver’s social support had a positive predictive effect on the patient’s self-disclosure and benefit finding (*β* = 0.179, *p* = 0.002; *β* = 0.148, *p* = 0.011).

In this study, a bias-corrected Bootstrap method was used to generate 5,000 repeated samples and calculate a 95% confidence interval to assess the mediating role of self-disclosure in the relationship between social support and benefit finding. If the confidence interval does not include 0, the mediating effect is confirmed. The results indicate that both patients’ and caregivers’ social support have statistically significant indirect effects on benefit finding through self-disclosure (*β* = 0.106, 95% *CI* = 0.055–0.156; *β* = 0.072, 95% *CI* = 0.029–0.115), indicating that the actor mediation effect is significant. A statistically significant indirect path was found linking caregivers’ social support to patients’ benefit finding through patients’ self-disclosure (*β* = 0.058, 95% *CI* = 0.015–0.102), indicating that the partner mediation effect is significant. See Table 4.

**Table 4:** Actor-partner mediation effects of sense of coherence, self-disclosure, and benefit finding in stroke patients and their caregivers.

Effect	Paths	*β* (standardized)	*SE*	*p*	95% *CI*
Patient actor effect					
Total effect	Patient social support→Patient benefit finding	0.287	0.046	<0.001	(0.197, 0.378)
Direct effect	Patient social support→Patient benefit finding	0.188	0.044	<0.001	(0.102, 0.274)
Indirect effect	Patient social support→Patient self-disclosure→Patient benefit finding	0.106	0.026	<0.001	(0.055, 0.156)
Indirect effect	Patient social support→Caregiver self-disclosure→Patient benefit finding	−0.006	0.008	0.417	(−0.021, 0.009)
Caregiver actor effect					
Total effect	Caregiver social support→Caregiver benefit finding	0.267	0.044	<0.001	(0.181, 0.352)
Direct effect	Caregiver social support→Caregiver benefit finding	0.171	0.039	<0.001	(0.094, 0.247)
Indirect effect	Caregiver social support→Caregiver self-disclosure→Caregiver benefit finding	0.072	0.022	0.001	(0.029, 0.115)
Indirect effect	Caregiver social support→Patient self-disclosure→Caregiver benefit finding	0.024	0.013	0.075	(−0.002, 0.050)
Patient partner effect					
Total effect	Caregiver social support→Patient benefit finding	0.183	0.056	0.001	(0.073, 0.294)
Direct effect	Caregiver social support→Patient benefit finding	0.148	0.058	0.011	(0.034, 0.262)
Indirect effect	Caregiver social support→Caregiver self-disclosure→Patient benefit finding	−0.023	0.016	0.152	(−0.055, 0.009)
Indirect effect	Caregiver social support→Patient self-disclosure→Patient benefit finding	0.058	0.022	0.009	(0.015, 0.102)
Caregiver partner effect					
Total effect	Patient social support→Caregiver benefit finding	0.286	0.054	<0.001	(0.180, 0.392)
Direct effect	Patient social support→Caregiver benefit finding	0.224	0.057	<0.001	(0.111, 0.336)
Indirect effect	Patient social support→Patient self-disclosure→Caregiver benefit finding	0.043	0.023	0.059	(−0.002, 0.089)
Indirect effect	Patient social support→Caregiver self-disclosure→Caregiver benefit finding	0.019	0.017	0.269	(−0.015, 0.053)

## 4 Discussion

Previous studies have largely examined the relationship between social support and benefit finding from a single perspective (46, 47). This study adopts a dyadic perspective—focusing on both stroke patients and their caregivers—to explore the actor-partner effects of social support on benefit finding, as well as the mediating role of self-disclosure in this relationship.

This study included a total of 278 pairs of stroke patients and their caregivers. The total score for benefit finding among stroke patients was (65.20 ± 8.41) points, with an average score per item of (2.96 ± 0.38) points; compared to the median item score of 3, this was at a slightly below-average level. The total score for benefit finding among caregivers was (71.57 ± 9.90) points, with an average item score of (3.25 ± 0.45) points, which was at a slightly above-average level. The study results show that the total benefit finding score for stroke patients was significantly lower than that of caregivers, which is consistent with the findings of Ding et al. (48). This may be because stroke patients not only suffer physically from disease-related complications (5) but also have a higher incidence of psychological problems (49); the immense physical and psychological stress may hinder their perception of the benefits of the disease (23).

The actor effect indicates that social support for both stroke patients and their caregivers can positively predict their own benefit finding. As an external resource for individuals coping with stressful events, social support can mitigate the negative effects of stress, serve as an emotional buffer, and help patients discover the positive aspects of their illness (50). For patients, social support can alleviate negative emotions arising from the illness, enabling them to cope more proactively and positively with adverse effects and to perceive the positive changes brought about by the illness (27). For caregivers, social support can mitigate the impact of stressful traumatic events, helping them face stress and difficulties more positively and, in turn, perceive their own positive growth (51).

Social support for stroke patients and their caregivers can positively predict self-disclosure. Research has found (52) that social support can increase an individual’s sense of security, thereby enhancing the level of self-disclosure. A supportive social environment can provide emotional comfort and a sense of security, which in turn encourages individuals to open up to others and express their true thoughts and emotions. Research has also found that self-disclosure among stroke patients and their caregivers can positively predict benefit finding. Self-disclosure helps rebuild cognitive structures, reduces the negative impact of traumatic events on individuals, and promotes their recovery and positive development (53). For patients, self-disclosure can enhance their sense of control over the disease, positively promoting mental health (54), and also helps them release negative emotions and reduce psychological distress, thereby yielding benefits (55).

The partner effect indicates that social support for stroke patients and their caregivers can positively predict each other’s benefit finding. This may be because patient support often helps share caregiving responsibilities, alleviates caregivers’ stress, and enables them to experience more benefits during the caregiving process; meanwhile, when caregivers receive adequate external support, their negative emotions are alleviated, and the quality of care improves, thereby providing patients with a better treatment and rehabilitation environment, which in turn increases the benefits patients perceive from their illness. Furthermore, patients’ self-disclosure positively predicts caregivers’ benefit finding, whereas caregivers’ self-disclosure does not predict patients’ benefit finding. This may be because patients’ self-disclosure serves as a bridge for caregivers to understand and support them; by listening to patients’ disclosures, caregivers can gain insight into their inner thoughts, strengthen emotional resonance, and recognize the benefits arising from the illness (56).

The mediation analysis reveals that social support for stroke patients and their caregivers is associated with benefit finding through self-disclosure; strong social support can encourage individuals to express their thoughts more freely and perceive positive meaning in their illness. However, there are differences in the effect sizes of their respective mechanisms of action. This is because there are fundamental differences in the identities of patients and caregivers, as well as in their sources of stress and resource needs, which result in varying degrees of effectiveness in benefit finding through social support and self-disclosure. Patients face physical and psychological harm caused by the disease itself, while caregivers primarily endure indirect caregiving stress. As the direct bearers of the illness, patients receive more diverse forms of social support, including financial and emotional support (57). In particular, financial support directly alleviates the burden of their illness, enabling them to release negative emotions through expressive writing or confiding in others, which ultimately promotes benefit finding in their illness. The social support received by caregivers consists primarily of intangible support, such as emotional regulation, which can effectively alleviate caregiving stress and burden (58); this type of support empowers them to express their inner thoughts and thereby discover the benefits of the caregiving process.

Social support for caregivers may be linked to patients’ self-disclosure and benefit finding; this pathway provides empirical support for the theory of interpersonal interdependence (19). This study found that, in addition to being directly associated with benefit finding, caregivers’ social support can also indirectly enhance patients’ benefit finding through patients’ self-disclosure. This finding reveals the cross-role mechanism of “caregiver resources → patient expression → patient benefit” in the stroke dyad, expands the theoretical implications of social support and benefit finding in a dyadic context, and provides a new entry point for clinical interventions by healthcare professionals.

## 5 Limitations

This study has several limitations. First, as a cross-sectional study, it cannot establish causal relationships among social support, self-disclosure, and benefit finding. Future longitudinal studies could further explore the causal relationships among these variables and, when combined with qualitative research, examine the relationships between them at different points in time. Second, the researchers selected patients and caregivers from only one hospital as study participants, which introduces selection bias and may limit the generalizability of the findings beyond the context of this study. Future studies should include participants from different time periods and conduct multicenter research. Third, the data in this study are based on self-reports from the study participants and are therefore subjective; they may be subject to reporting bias and may be influenced by confounding factors such as patients’ functional status and caregiver burden. In future studies, subjective assessments could be supplemented with objective clinical and physiological indicators and professional clinical evaluations to control for confounding factors, thereby further enhancing the reliability of the data and their clinical applicability.

## 6 Conclusion

From a dyadic perspective of interdependence between stroke patients and their caregivers, this study found an association between social support and benefit finding, and that this association is mediated by self-disclosure. This suggests that healthcare professionals can use assessment tools to identify patients and caregivers with low levels of social support and self-disclosure, and implement targeted measures to help them cope with the stress caused by the disease and derive benefits from it. In the future, healthcare professionals will be able to use systematic, multidimensional intervention strategies to help stroke patients and their caregivers expand their support networks and build collaborative support systems involving family, peers, and the community; create a safe and inclusive communication environment for stroke patients and their caregivers, encouraging both parties to express their inner thoughts moderately and authentically; simultaneously, they can integrate benefit finding interventions into daily nursing practice, such as narrative communication and meaning reconstruction, can be incorporate positive psychological interventions throughout the entire nursing process, including health education, rehabilitation guidance, and discharge planning, thereby enhancing the scalability and sustainability of nursing practice.

## Data Availability

The raw data supporting the conclusions of this article will be made available by the authors, without undue reservation.

## References

[1] Group RoSCiCW. Brief report on stroke center in China, 2022. Chin J Cerebrovasc Dis. (2024) 21:565–76. doi: 10.3969/j.issn.1672-5921.2024.08.009

[2] Collaborators GS. Global, regional, and national burden of stroke and its risk factors, 1990–2019: a systematic analysis for the global burden of disease study 2019. Lancet Neurol. (1990) 20:795–820. doi: 10.1016/s1474-4422(21)00252-0, 34487721 PMC8443449

[3] Feigin VL Brainin M Norrving B Martins S Sacco RL Hacke W . World stroke organization (WSO): global stroke fact sheet 2022. Int J Stroke. (2022) 17:18–29. doi: 10.1177/17474930211065917, 34986727

[4] Group RoSPaTiCW. Brief report on stroke prevention and treatment in China, 2021. Chin J Cerebrovasc Dis. (2023) 20:783–93. doi: 10.3969/j.issn.1672-5921.2023.11.009

[5] Lekoubou A Nguyen C Kwon M Nyalundja AD Agrawal A. Post-stroke everything. Curr Neurol Neurosci Rep. (2023) 23:785–800. doi: 10.1007/s11910-023-01308-9, 37837566

[6] El Husseini N Katzan IL Rost NS Blake ML Byun E Pendlebury ST . Cognitive impairment after ischemic and Hemorrhagic stroke: a scientific statement from the American Heart Association/American Stroke Association. Stroke. (2023) 54:e272–91. doi: 10.1161/str.0000000000000430, 37125534 PMC12723706

[7] Yuliana S Yu E Rias YA Atikah N Chang HJ Tsai HT. Associations among disability, depression, anxiety, stress, and quality of life between stroke survivors and their family caregivers: an actor-partner interdependence model. J Adv Nurs. (2022) 79:135–48. doi: 10.1111/jan.15465, 36257927

[8] Almhdawi KA Alazrai A Kanaan S Shyyab AA Oteir AO Mansour ZM . Post-stroke depression, anxiety, and stress symptoms and their associated factors: a cross-sectional study. Neuropsychol Rehabil. (2020) 31:1091–104. doi: 10.1080/09602011.2020.1760893, 32419606

[9] Zhao J Zeng Z Yu J Xu J Chen P Chen Y . Effect of main family caregiver’s anxiety and depression on mortality of patients with moderate-severe stroke. Sci Rep. (2021) 11:2747. doi: 10.1038/s41598-021-81596-8, 33531519 PMC7854741

[10] Xu Q Ma J Zhang Y Gan J. Family resilience and social support as mediators of caregiver burden and capacity in stroke caregivers: a cross-sectional study. Front Psychol. (2024) 15:15. doi: 10.3389/fpsyg.2024.1435867, 39698379 PMC11653184

[11] Zhong X Chang Z Zhou C. The effectiveness of a brief cognitive Behavioral stress management on benefit finding and quality of life in patients with colorectal cancer. Chin. J Clin Psychol. (2022) 30:1000. doi: 10.16128/j.cnki.1005-3611.2022.04.048

[12] Helgeson VS Reynolds KA Tomich PL. A meta-analytic review of benefit finding and growth. J Consult Clin Psychol. (2006) 74:797–816. doi: 10.1037/0022-006x.74.5.797, 17032085

[13] Tang Y Zhang X Yang Q Zhang X. Mediating role of cognitive appraisal and benefit finding in relationship between fear of progression and quality of life in patients with chronic heart failure. Chin Ment Health J. (2025) 39:943–8. doi: 10.3969/j.issn.1000-6729.2025.11.03

[14] Wang X Xie J Wang Y Zhang Y. Relationship between disease sense of benefit, big five personality and quality of life in breast cancer patients. Chin Nurs Res. (2024) 38:3423–8. doi: 10.12102/j.issn.1009-6493.2024.19.007

[15] Liu Y-t Zhang S-x Xiang D-d Ge S Liu Z-w Fan B-y . Perceived social support mediates the relationship between benefit finding and depressive symptoms among young and middle-aged stroke survivors and their spouse caregivers. J Cardiovasc Nurs. (2025) 40:E257–66. doi: 10.1097/jcn.0000000000001161, 39620644

[16] Chang M-W Wang AW-T Chang C-S. The role of perceived benefits in buffering gastrointestinal-symptom burden among post-operative colorectal Cancer patients: a six-month longitudinal study. Cancer. (2025) 17:2934. doi: 10.3390/cancers17172934, 40941031 PMC12428437

[17] Zhao Q Li M Zhang J Xu X Zhao H. Research progress of benefits finding for family caregivers of stroke patients. Chin J Pract Nurs. (2023) 39:1031–5. doi: 10.3760/cma.j.cn211501-20220408-01057

[18] Lyons KS Lee CS. The theory of dyadic illness management. J Fam Nurs. (2018) 24:8–28. doi: 10.1177/1074840717745669 29353528

[19] Kelley HH Thibaut JW. Interpersonal Relations: A Theory of Interdependence. New York: John Wiley & Sons (1978).

[20] Shi H Gu Z Liao Y Wan X Ji L. Mediating effect of family resilience on the relationship between activities of daily living of stroke patients and the burden of the primary caregivers. J Nurs Sci. (2023) 38:53–6. doi: 10.3870/j.issn.1001-4152.2023.17.053

[21] Yuan H You J Huang J Zhao W Jin X Xu W. An actor-partner interdependence mediation model for assessing the association between dyadic coping and post-traumatic growth in dyads of patients with stroke and their spouses: cross-sectional study. BMC Psychol. (2026) 14:705. doi: 10.1186/s40359-026-04497-3, 41928330 PMC13170005

[22] Rago C De Maria M Vellone E Alvaro R Zeffiro V Virgolesi M . Associations between dyadic spirituality, caregiver burden, disability, and health-related quality of life in stroke survivor–caregiver dyads: a dyadic structural equation modelling study. Eur J Cardiovasc Nurs. (2026) 25:756–65. doi: 10.1093/eurjcn/zvag016, 41553920

[23] Zhang J Li H Chen J Jia A Zhang Y Wang D . Latent profile analysis for benefit finding in ischemic stroke patients: a cross-sectional study. Acta Psychol. (2025) 260:105500. doi: 10.1016/j.actpsy.2025.105500, 40897144

[24] Lou C Xiaoxia D Wang H OuYang H Liu Y Chen T. Potential profiles of caregivers' benefit finding in ischaemic stroke: the role of personality traits and coping styles. Acta Psychol. (2025) 253:104734. doi: 10.1016/j.actpsy.2025.104734, 39837073

[25] Sun Q Mei Y Song Y Huang Z Liu Z Duan B . A study on dyadic relationship between benefit finding and positive psychological capital in stroke patients and their spouses. Chin J Nurs. (2024) 59:2116–22. doi: 10.3761/j.issn.0254-1769.2024.17.010

[26] Zhao M Na N Xing N Zhu H Wu G Xu G . The impact of social support on benefit finding among patients with advanced lung cancer and their caregivers: based on actor-partner interdependence mediation model. Support Care Cancer. (2024) 32:287. doi: 10.1007/s00520-024-08435-0, 38619660

[27] Liu F Dong J Yi J Ma S Zou J Dai Y. A study on the discovery level of benefit finding for elderly patients with hypertension and its impact pathways. J Nurs Adm. (2025) 25:863–8. doi: 10.3969/j.issn.1671-315x.2025.10.007

[28] Qian Y Zheng L Li J. Benefit finding and its influencing factors in patients with thyroid cancer: a retrospective study. Asia Pac J Oncol Nurs. (2024) 11:100584. doi: 10.1016/j.apjon.2024.100584, 39411575 PMC11472082

[29] Li L Chen H. Relationship of self-disclosure with personality characteristics, loneliness and mental health in college students. Chin J Tissue Eng Res. (2004) 33:7568–70. doi: 10.3321/j.issn:1673-8225.2004.33.018

[30] Chen J Cheng H Wei Q Zhong C Xu Y Xia y . Self-disclosure intervention for patients undergoing chemotherapy following breast cancer surgery. J Nurs Sci. (2022) 37:86–9. doi: 10.3870/j.issn.1001-4152.2022.14.086

[31] Zhu Y Cai N Wang Z Li J Guo T He J . Perceived social support and psychosocial adaptation in patients with chronic skin diseases: the mediating role of self-disclosure. Front Psych. (2026) 16:1731560. doi: 10.3389/fpsyt.2025.1731560, 41788471 PMC12956801

[32] Guo C Zhou C Teng Z Li L. Social support mediates the relationship between self-disclosure and benefit finding in patients with permanent intestinal ostomy due to colorectal cancer. J Colorectal Anal Surg. (2023) 29:292–8. doi: 10.19668/j.cnki.issn1674-0491.2023.03.020

[33] Taylor SE Stanton AL. Coping resources, coping processes, and mental health. Annu Rev Clin Psychol. (2007) 3:377–401. doi: 10.1146/annurev.clinpsy.3.022806.091520, 17716061

[34] Neurology CSo, Society CS. Chinese guidelines for diagnosis and treatment of acute ischemic stroke 2018. Chin J Neurol. (2018) 51:666–82. doi: 10.3760/cma.j.issn.1006-7876.2018.09.004

[35] Neurology CSo, Society CS. Chinese guidelines for diagnosis and treatment of acute intracerebral hemorrhage 2019. Chin J Neurol. (2019) 52:994–1005. doi: 10.3760/cma.j.issn.1006-7876.2019.12.003

[36] Arakd A. APIMPowerR:An interactive tool for Actor-Partner Interdependence Model power analysis. (2025). Available online at: http://robert-a-ackerman.shinyapps.io/APIMPowerRdis/.

[37] Xiao S. Theoretical basis and research applications of the social support rating scale. J Clin Psychiatry. (1994) 02:98–100.

[38] Kahn JH Hessling RM. Measuring the tendency to conceal versus disclose psychological distress. J Soc Clin Psychol. (2001) 20:41–65. doi: 10.1521/jscp.20.1.41.22254

[39] Li X. Study on the Relationship Among Adult Attachment, Self-Disclosure and Depression Status of College Students [Master. Shijiazhuang: Hebei Normal University (2009).

[40] Antoni MH Lehman JM Kilbourn KM Boyers AE Culver JL Alferi SM . Cognitive-behavioral stress management intervention decreases the prevalence of depression and enhances benefit finding among women under treatment for early-stage breast cancer. Health Psychol. (2001) 20:20–32. doi: 10.1037//0278-6133.20.1.20, 11199062

[41] Weaver KE Llabre MM Lechner SC Penedo F Antoni MH. Comparing unidimensional and multidimensional models of benefit finding in breast and prostate cancer. Qual Life Res. (2008) 17:771–81. doi: 10.1007/s11136-008-9348-z, 18500579

[42] Liu Z Zhang L Gudenkauf L. Cross-cultural adaptation of the benefit finding scale (BFS) in Chinese cancer patients. Chin J Nurs. (2015) 50:561–6. doi: 10.3761/j.issn.0254-1769.2015.05.010

[43] Liu Z Xiang D Ge S Mei Y Zhang Z Chen S . Trajectories of dyadic quality of life in young to middle-aged stroke couples: a longitudinal study. Qual Life Res. (2024) 34:669–81. doi: 10.1007/s11136-024-03839-1, 39630394

[44] Bian J Zhang L Liu Z Ni T Li Y. Reliability and validity of the revised Chinese version of benefit finding scale in family caregivers of cancer patients. Chin Gen Pract. (2018) 21:2091–6. doi: 10.3969/j.issn.1007-9572.2018.00.222

[45] Wang R Zhang Y lv X Wu D Wu M. Reliability and validity test of the benefit finding scale in caregivers of ischemic stroke patients. Chin Nurs Res. (2020) 34:2655–9. doi: 10.12102/j.issn.1009-6493.2020.15.007

[46] Rong H Yin M Ren P Li Y Qu H Chen X. Spirituality as a mediator between social support and benefit finding among advanced Cancer patients. Cancer Nurs. (2023) 46:E230–7. doi: 10.1097/ncc.0000000000001134, 36461932 PMC10289222

[47] Jiang Y Hu N Chang T. The mediating role of hope in the link between social support and benefit finding among caregivers of children undergoing liver transplantation. J Pediatr Nurs. (2025) 85:722–30. doi: 10.1016/j.pedn.2025.10.008, 41135364

[48] Ding Z Fan Y Zhong G Zhang X Li X Qiao Y . A dyadic analysis of family adaptation among breast cancer patients and their spouses based on the framework of family stress coping theory. Front Public Health. (2024) 12:25. doi: 10.3389/fpubh.2024.1453830, 39655260 PMC11625673

[49] Li Y Zhong G Liao Q Rong C Yao R. Investigation and analysis report on the psychological status of stroke rehabilitation patients. Psychologies Magazine. (2025) 20:206–208+212. doi: 10.19738/j.cnki.psy.2025.22.055

[50] Qiu X Zhang K Zhang Y Sun L. Benefit finding and related factors of patients with early-stage Cancer in China. Int J Environ Res Public Health. (2022) 19:4284. doi: 10.3390/ijerph19074284, 35409965 PMC8999120

[51] Zhang H Li Z Luo W Huang H Lin Y Zhang J. Systematic review of mixed studies on influencing factors of benefit finding in stroke caregivers. Chinese General Practice Nursing. (2025) 23:1564–70. doi: 10.12104/j.issn.1674-4748.2025.08.040

[52] Zhen R Quan L Zhou X. How does social support relieve depression among flood victims? The contribution of feelings of safety, self-disclosure, and negative cognition. J Affect Disord. (2018) 229:186–92. doi: 10.1016/j.jad.2017.12.087, 29324365

[53] Schlosser AE. Self-disclosure versus self-presentation on social media. Curr Opin Psychol. (2020) 31:1–6. doi: 10.1016/j.copsyc.2019.06.025, 31357096

[54] Zhang X Huang T Sun D Liu M Wang Z. Illness perception and benefit finding of thyroid Cancer survivors: a chain mediating model of sense of coherence and self-disclosure. Cancer Nurs. (2025) 48:437–44. doi: 10.1097/ncc.0000000000001347, 38527098

[55] Liu X Cheng X Yang F Tong H. The mediating roles of intimate relationships and constructive communication in the association between self-disclosure and benefit finding among patients with colorectal cancer. Asia Pac J Oncol Nurs. (2025) 12:100758. doi: 10.1016/j.apjon.2025.100758, 40756132 PMC12318292

[56] Zhang Z Li X Wang Z Yang Y Zhu D Jiang X . Analysis of influencing factors and impact path of benefit finding in patients with cervical cancer and their spouses. Chin J Nurs. (2024) 59:2214–21. doi: 10.3761/j.issn.0254-1769.2024.18.005

[57] Wu XJ Ke K Liu H Zhan SP Wang L He JF. Social isolation in the young and middle-aged patients with stroke: role of social support, family resilience and hope. Front Psych. (2025) 16:1499186. doi: 10.3389/fpsyt.2025.1499186, 40012713 PMC11861369

[58] Zhang Y Ma J Chen L Chen H Xu Y Xu H . Perceived social support, caregiver capacity, and socioeconomic determinants mediating pathways to family resilience in Chinese stroke survivors: a cross-sectional study. BMC Nurs. (2025) 24:1180. doi: 10.1186/s12912-025-03826-y, 41013521 PMC12465349

